# Evaluation of General Public Awareness, Knowledge, and Attitude Towards Attention-Deficit/Hyperactivity Disorder in the Riyadh Region of Saudi Arabia

**DOI:** 10.7759/cureus.91607

**Published:** 2025-09-04

**Authors:** Salahuddin Khan, Ammar Y Al Qahtani, Abdulrahman K Aburas, Sultan H AlKhashan, Fahad A Giraud, Khaled M Alolayan, Abdullah A AL Mohsen, Abdulaziz M Almegren, Mohammed I Albalawi

**Affiliations:** 1 Biochemistry, College of Medicine, Al Imam Mohammad Ibn Saud Islamic University, Riyadh, SAU; 2 Medicine, College of Medicine, Al Imam Mohammad Ibn Saud Islamic University, Riyadh, SAU

**Keywords:** and practices (kap), attention-deficit/hyperactivity disorder (adhd), attitude, attitudes, awareness, knowledge, riyadh, saudi arabia

## Abstract

Background

Attention‑deficit/hyperactivity disorder (ADHD) is a common neurodevelopmental disorder with a global prevalence of around 5% among children and adolescents. Despite increasing diagnoses, public knowledge about ADHD remains limited, contributing to stigma and delayed care. In Saudi Arabia, evidence on community awareness and attitudes is sparse.

Objective

This study assessed the awareness, knowledge, attitudes, and selected practices related to ADHD among adults in the Riyadh region of Saudi Arabia.

Methods

A cross‑sectional survey was conducted from July 2024 to January 2025 using a self‑administered online questionnaire distributed via WhatsApp. Adults ≥18 years residing in Riyadh and able to read Arabic or English were invited. The instrument included four demographic questions and 23 items assessing knowledge (multiple choice) and attitudes (Likert scale). Knowledge scores were categorized as poor (<50% of items correct), moderate (50-75%), or good (>75%). Attitude scores were classified as negative (<50%), neutral (50-75%), or positive (>75%). Practice questions on help‑seeking and information sources were analyzed descriptively. Data were analyzed using SPSS, and normality was assessed using the Kolmogorov-Smirnov test. Because knowledge and attitude scores were non‑normally distributed, Mann‑Whitney U and Kruskal-Wallis H tests were used to compare groups, and Spearman’s correlation was used to assess associations.

Results

A total of 573 adults participated in the survey (response above the calculated sample of 385). Most were aged 18-25 years (60.2%) and were male (63%). Approximately 75% had previously heard of ADHD, but only 23.9% recognized it as a lifelong condition. Although 79.4% and 77.1% correctly identified inattention and hyperactivity as core symptoms, respectively, only 42.4% recognized impulsivity. Genetic factors were acknowledged by 61.1%, and 80.5% endorsed behavioral and pharmacological management. The mean (± SD) knowledge score was 6.48 ± 1.39. Out of the 573 participants, 49.9% showed poor knowledge, 48.9% showed moderate knowledge, and only 1.2% showed good knowledge. The attitude domain mean score was 39.9 ± 4.19 (range 13-55); 36.8% had a positive attitude, 62.8% had a neutral attitude, and 0.3% had a negative attitude. Most respondents believed that treatment and parenting skills improved outcomes, yet many hesitated to support inclusion of ADHD children in mainstream schools. Regarding practice, 55.7% had sought information about ADHD, 33.7% knew where to seek help, and 43.6% had interacted with someone with ADHD. The most common information source was the internet (78.7%), followed by friends/family (35.1%). Knowledge and attitude scores were positively correlated (rs = 0.165, p < 0.001). Higher education, female gender, and previous exposure to ADHD were associated with higher knowledge and more positive attitudes (p < 0.05).

Conclusion

Despite widespread awareness of ADHD, substantial gaps exist in public understanding of its symptoms, chronicity, and etiology. Attitudes were generally neutral, with reluctance toward full educational inclusion. Targeted educational initiatives and public campaigns are necessary to improve knowledge and foster supportive attitudes toward individuals with ADHD in Saudi Arabia.

## Introduction

Neurodevelopmental disorders are a group of conditions that begin in early childhood and are characterized by developmental deficits affecting personal, social, academic, or occupational functioning [1]. Among these, attention-deficit/hyperactivity disorder (ADHD) is one of the most prevalent. According to the Diagnostic and Statistical Manual of Mental Disorders, Fifth Edition (DSM-5), ADHD is defined as “a persistent pattern of inattention and/or hyperactivity-impulsivity that interferes with functioning or development” [1]. The core symptoms include difficulty sustaining attention, hyperactivity, and impulsivity. For a formal diagnosis, symptoms must be present in at least two different settings, such as home and school, and cause significant impairment [1].

Globally, ADHD affects approximately 5-7% of children, making it one of the most common psychiatric conditions in childhood [2]. Importantly, evidence indicates that ADHD is not confined to childhood, as it often persists into adolescence and adulthood. In adults, hyperactivity may diminish, but difficulties in attention, executive functioning, and emotional regulation remain prominent [3,4]. The chronic nature of ADHD significantly impacts academic achievement, occupational performance, interpersonal relationships, and overall quality of life [3,5].

In Saudi Arabia and other Middle Eastern countries, prevalence studies have reported ADHD rates in children ranging from 3.4% to 9.2%, depending on diagnostic criteria and assessment tools used [6,7]. Despite its considerable burden, ADHD remains underdiagnosed and is often misunderstood by the general public, with misconceptions such as attributing the condition to poor parenting or dietary habits. This lack of accurate knowledge, combined with stigma, can delay early detection and appropriate treatment, thereby exacerbating long-term outcomes.

Although several studies have examined ADHD prevalence and teachers’ knowledge in Saudi Arabia [6,7], there remains limited evidence on knowledge, attitudes, and practices (KAP) regarding ADHD among the general adult population. Assessing these factors is essential, as public perceptions influence help-seeking behaviors, treatment adherence, and the broader social support available to affected individuals.

Therefore, the current study was conducted to evaluate the KAP related to ADHD among adults in Riyadh, Saudi Arabia, and to examine the association between sociodemographic characteristics and levels of awareness. This research is justified by the persistent misconceptions and low levels of awareness reported in prior studies, highlighting the urgent need for targeted educational interventions to enhance understanding, reduce stigma, and support early recognition and effective management of ADHD.

## Materials and methods

Study design and setting

This was a descriptive cross‑sectional study conducted in the Riyadh region of Saudi Arabia between July 2024 and January 2025. The study adhered to the Strengthening the Reporting of Observational Studies in Epidemiology (STROBE) guidelines. Participation was voluntary, and informed consent was obtained electronically before respondents accessed the survey.

Participants and sampling

Eligible participants were adults (≥ 18 years) residing in Riyadh and capable of reading Arabic or English. Individuals working in health‑related fields were not excluded because the aim was to capture general community perspectives. A minimum sample of 385 participants was calculated using the single‑proportion formula (assuming 50% expected adequate knowledge, 5% margin of error, and 95% confidence level). To improve representativeness and account for potential incomplete responses, the survey remained open until January 2025, yielding 573 complete responses.

Data collection instrument

Data were collected using a self‑administered questionnaire designed after reviewing relevant literature and previously validated KAP instruments. The final questionnaire contained 27 items divided into four sections: four demographic items (age, gender, occupation, and educational attainment), 23 items assessing knowledge and attitudes about ADHD, and several unscored questions about practices. The knowledge section comprised multiple‑choice and yes/no questions on core symptoms, causes, diagnosis, and treatment options. Each correct answer scored one point; incorrect or “not sure” responses scored zero. Attitudes were measured using a five‑point Likert scale (1 = strongly disagree to 5 = strongly agree) across statements addressing perceived seriousness, treatment efficacy, educational inclusion, and stigma. Summed attitude scores ranged from 13 to 65. Practices were explored through questions on help‑seeking, advocacy, and information sources; these items were reported descriptively and not included in the attitude score.

The questionnaire was prepared in English and then translated into Arabic by bilingual experts following forward and backward translation. A pilot test with 20 individuals ensured clarity and cultural appropriateness. Minor modifications were made based on feedback. Internal consistency of the knowledge and attitude scales was acceptable (Cronbach’s alpha = 0.86). The final survey was hosted on Google Forms and distributed via WhatsApp networks to maximize reach and minimize selection bias. Duplicate submissions were prevented by restricting responses to one per electronic device. The complete questionnaire (27 items) is provided in Appendix A.

Scoring and outcome measures

Knowledge scores ranged from 0 to 14. Scores were categorized as follows: poor knowledge (<50% of items correct), moderate knowledge (50-75%), and good knowledge (>75%). Attitude scores were interpreted similarly: negative (<50% of the maximum), neutral (50-75%), and positive (>75%). Practice outcomes, including whether participants had sought ADHD information, were aware of help services, and had previously interacted with an individual with ADHD, were summarized in proportions.

Statistical analysis

Data were analyzed using SPSS version 28 (IBM Corp., Armonk, NY). Continuous variables were summarized as means ± standard deviations, and categorical variables were summarized as frequencies and percentages. The Kolmogorov-Smirnov test was used to assess normality of knowledge and attitude scores; both distributions were non‑normal. Consequently, non‑parametric tests were used to evaluate differences between groups: Mann‑Whitney U tests were used to compare two groups (e.g., gender), and Kruskal‑Wallis H tests were used to compare more than two groups (e.g., education levels). Spearman’s rank correlation coefficient was used to examine the relationship between knowledge and attitude scores. A p‑value of <0.05 was considered statistically significant.

Ethical considerations

This study was reviewed and approved by the Institutional Review Board of Al‑Imam Muhammad Ibn Saud Islamic University (IRB registration HAPO‑01‑R‑0011; project number 673/2024). The protocol received full board approval on August 28, 2024. Data were collected anonymously, stored securely, and used solely for research purposes.

## Results

A total of 573 participants completed the survey (response rate not calculable due to open distribution). The majority were between 18 and 25 years of age (n = 345, 60.2%), followed by 26-30 years (n = 112, 19.5%) and >30 years (n = 116, 20.2%). Males represented 63.0% (n = 361) of the sample, and 71.2% (n = 408) were students or employed in the private or public sectors. Approximately 75.2% (n = 431) reported having heard of ADHD previously. Most participants held a bachelor’s degree or higher (n = 336, 58.6%) (Table 1).

**Table 1:** Sociodemographic characteristics of participants (n=573) ADHD, attention-deficit/hyperactivity disorder

Study data	N (%)
Age group	
18–25 years	345 (60.2%)
26–30 years	112 (19.5%)
>30 years	116 (20.2%)
Gender	
Male	361 (63.0%)
Female	212 (37.0%)
Occupation	
Student	214 (37.3%)
Employed	191 (33.3%)
Unemployed	168 (29.3%)
Educational level	
High school	96 (16.8%)
College level	290 (50.6%)
College graduate	71 (12.4%)
Postgraduate	116 (20.2%)
Previously heard ADHD	
Yes	431 (75.2%)
No	142 (24.8%)

Assessment of knowledge about ADHD

Table 2 summarizes the responses to knowledge items. Most respondents correctly identified inattention (79.4%) and hyperactivity (77.1%) as ADHD symptoms, whereas fewer recognized impulsivity (42.4%). Regarding onset, 79.6% believed that ADHD manifests during preschool years, and none selected adulthood. Only 23.9% acknowledged that ADHD often persists throughout life. Genetic factors were recognized by 61.1%, whereas smaller proportions endorsed environmental or parenting causes. Most participants (80.5%) correctly believed that management involves multi‑modal strategies such as behavioral therapy, parenting support, and medication. The mean knowledge score was 6.48 ± 1.39 (range 0-14). Overall, 286 (49.9%) participants demonstrated poor knowledge, 280 (48.9%) demonstrated moderate knowledge, and only seven (1.2%) demonstrated good knowledge. The distribution of responses to knowledge items is presented in Table 2; the full questionnaire is available in Appendix A.

**Table 2:** Assessment of the knowledge about ADHD (n=573) ADHD, attention-deficit/hyperactivity disorder

Knowledge items	N (%)
Knowledge about the main symptoms of ADHD	
1. Inattention [yes]	455 (79.4%)
2. Hyperactivity [yes]	442 (77.1%)
3. Impulsivity [yes]	243 (42.4%)
4. Aggression [yes]	104 (18.2%)
At what age do you think ADHD is usually diagnosed?	
5. Preschool [yes]	456 (79.6%)
6. Primary school [yes]	98 (17.1%)
7. Adolescence [yes]	19 (03.3%)
8. Adulthood [yes]	0
9. Do you believe ADHD is a lifelong condition? [yes]	137 (23.9%)
Causes of ADHD	
10. Genetic factors [yes]	350 (61.1%)
11. Poor parenting [yes]	180 (31.4%)
12. Diet [yes]	170 (29.7%)
13. Environmental factors [yes]	281 (49.0%)
14. Correct management of ADHD [The right way to manage is drastic changes such as lifestyle changes, cognitive behavioral therapy, medication]	461 (80.5%)
Total knowledge score (mean ± SD)	6.48 ± 1.39
Level of knowledge	
Poor	286 (49.9%)
Moderate	280 (48.9%)
Good	7 (01.2%)

Assessment of attitude toward ADHD

As shown in Table 3, the top three attitudes with the highest mean percentage rating were as follows: "Do you believe children with ADHD can lead a normal life with proper treatment?" (85.4%), followed by "How would the enhancement of parenting skills contribute to the well-being of their child?" (85.2%), and "How beneficial do you consider social skills training to be for children diagnosed with ADHD?" (81.6%). In contrast, the attitude mean percentage rating was lowest in the statement, "Do you think children with ADHD should be in regular schools or special schools?" (47%). The overall mean attitude score was 39.9 (SD 4.19). Based on classification thresholds, 211 (36.8%) participants had a positive attitude, 360 (62.8%) had a neutral attitude, and two (0.3%) had a negative attitude. “Participants’ responses to the attitude items are summarized in Table 3; corresponding questionnaire items are listed in Appendix A.

**Table 3:** Assessment of the attitude about ADHD (n=573) ADHD, attention-deficit/hyperactivity disorder

Attitude items	Item	Mean score	Mean (%)
	Score	Mean ± SD	
1. Do you believe children with ADHD can lead a normal life with proper treatment? [1=strongly disagree to 5=strongly agree]	5	4.27 ± 0.77	85.40%
2. To what extent would you rely on an individual with ADHD to complete a critical task? [1=I can’t rely to 4=I can completely rely]	4	2.88 ± 0.83	72.00%
3. What are your emotional responses and feelings regarding your child consulting a psychiatrist for an ADHD assessment and receiving a potential diagnosis? [1=negative to 3=positive]	3	2.27 ± 0.81	75.70%
4. How would the enhancement of parenting skills contribute to the well-being of their child? [1=not at all to 5=very significantly]	5	4.26 ± 0.89	85.20%
5. How beneficial do you consider social skills training to be for children diagnosed with ADHD? [1=not at all to 5=very significantly]	5	4.08 ± 0.87	81.60%
6. Should children with ADHD be treated differently within the household context? [1=no to 4=yes, they should be treated with more understanding and support]	4	3.20 ± 1.07	80.00%
7. To what extent do familial issues contribute to the manifestation of ADHD in children? [1=not at all to5=very significantly]	5	3.31 ± 1.05	66.20%
8. Do you think ADHD is potentially a consequence of a child's lack of effort in controlling their behavior? [1=strongly disagree to 5=strongly agree]	5	3.12 ± 1.02	62.40%
9. How might contemporary social media usage impact a child's attention span negatively? [1=not at all to 5=very significantly]	5	3.76 ± 0.97	75.20%
10. Do the side effects of ADHD medications outweigh their therapeutic benefits? [1=strongly agree to 5=strongly disagree]	5	3.05 ± 0.79	61.00%
11. Do you think children with ADHD should be in regular schools or special schools? [0=not sure; 1=regular school; 2= special school]	2	0.94 ± 0.67	47.00%
12. Would you feel uneasy if your child's classmate had ADHD? [0=not sure; 1=yes; 2=no]	2	1.37 ± 0.81	68.50%
13. Do you think medication is an effective treatment for ADHD? [1=strongly disagree to 5=strongly agree]	5	3.50 ± 0.88	70.00%
Total attitude score	55	39.9 ± 4.19	72.50%
Level of attitude, N (%)			
Negative	-	02 (0.30%)	-
Neutral	-	360 (62.8%)	-
Positive	-	211 (36.8%)	-

**Figure 1:**
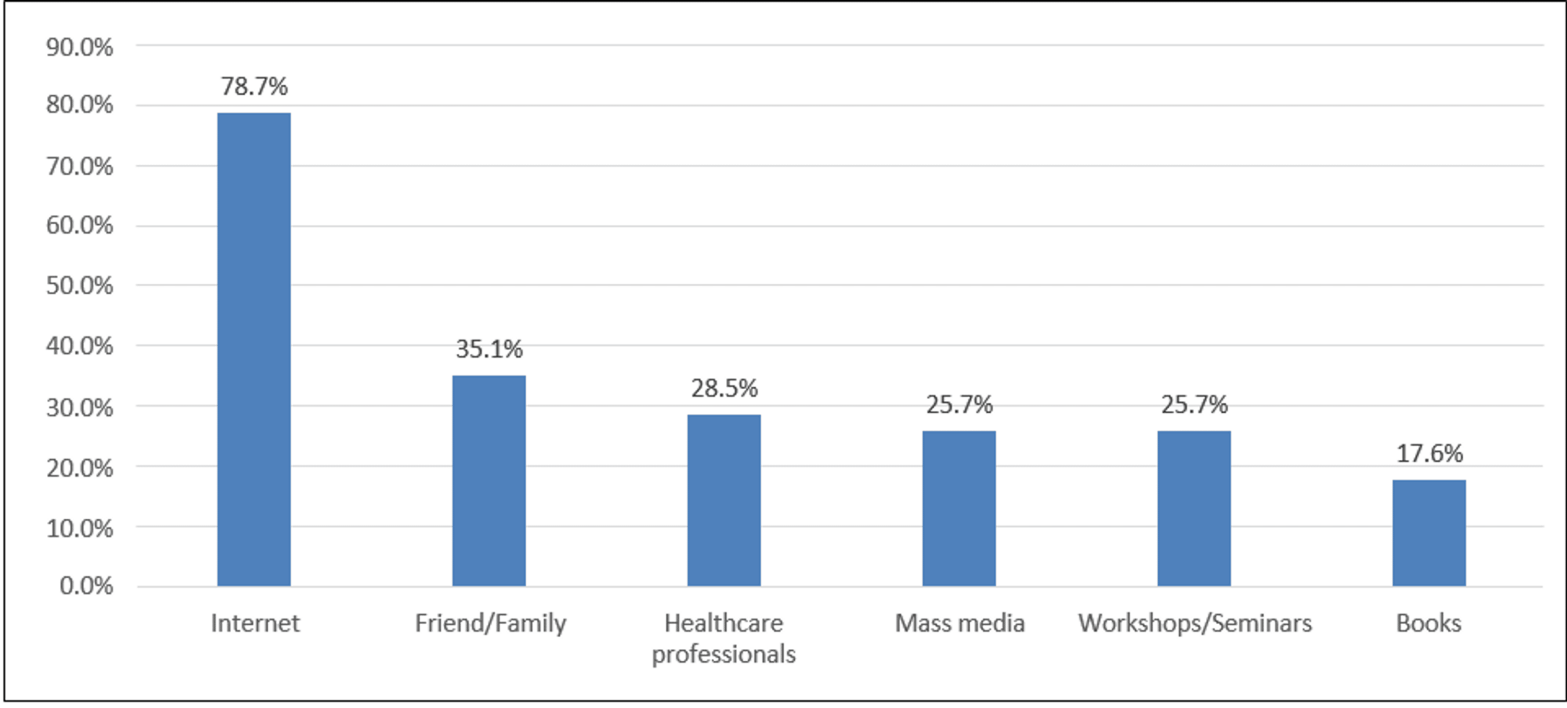
Source of ADHD information ADHD, attention-deficit/hyperactivity disorder

As shown in Table 4, 55.7% of participants reported having sought information about ADHD from at least one source, most commonly the internet (78.7%), followed by friends or family (35.1%), healthcare professionals (28.5%), media (14.3%), and books (11.1%) (Figure 1; Appendix Q19). Approximately one-third (33.7%) of participants knew of places to seek help if they suspected their child had ADHD. Overall, 45% would give medication to their children with ADHD. Of those who use medication for their children, 24.4% were aware of medication side effects. Only 5.4% engage regularly in activities or reading materials about ADHD. The proportion of participants who dealt with ADHD patients was 43.6%, with relative being the most common (54.4%). In addition, only 25.7% knew how to support their ADHD child's needs at school. Practice-related findings are shown in Table 4, with the relevant questionnaire items detailed in Appendix A.

**Table 4:** Assessment of practice about ADHD (n=573) ADHD, attention-deficit/hyperactivity disorder

Practice items	N (%)
Have you ever sought information about ADHD from any source?	
Yes	319 (55.7%)
No	254 (44.3%)
Are you familiar with places that would help if you had a suspected ADHD child?	
Yes	193 (33.7%)
No	380 (66.3%)
If you had a child with ADHD, would you consider using medication as part of the treatment?	
Yes	258 (45.0%)
No	92 (16.1%)
Not sure	223 (38.9%)
If yes, are you aware of the side effects? (n=258)	
Yes	63 (24.4%)
No	195 (75.6%)
How often do you engage in activities or read materials to increase your understanding of ADHD?	
Regularly	31 (05.4%)
Occasionally	148 (25.8%)
Rarely	219 (38.2%)
Never	175 (30.5%)
Have you ever dealt with an ADHD individual?	
Yes	250 (43.6%)
No	190 (33.2%)
Not sure	133 (23.2%)
If yes, who? (n=250)	
A relative	136 (54.4%)
A friend	85 (34.0%)
At work	29 (11.6%)
If your child is suffering from ADHD, do you know how to advocate your child's needs in school?	
Yes	147 (25.7%)
No	185 (32.3%)
Not sure	241 (42.1%)

Among those who sought ADHD information (N=319), the most common source of ADHD information was the internet (78.7%), followed by friends/family (35.1%) and healthcare professionals (28.5%) (Figure 1).

**Figure 2:**
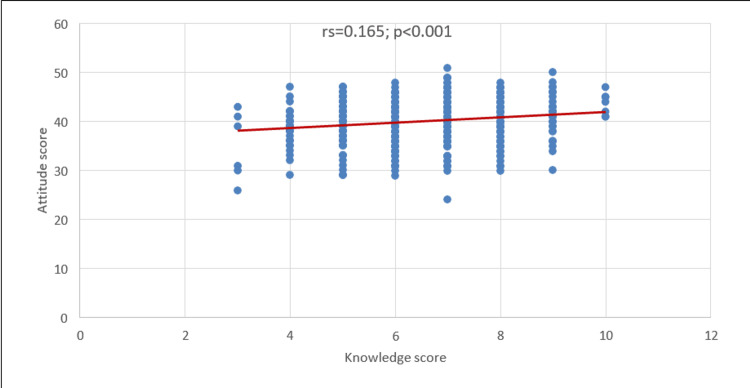
Correlation between knowledge and attitude scores

Figure 2 shows a significant positive correlation between knowledge and attitude score (rs=0.165; p<0.001), suggesting that the increase in knowledge score correlates with the increase in attitude score.

Investigating variations in knowledge and attitude

Scores found that a higher knowledge score was more associated with having heard of ADHD (Z=2.261; p=0.024), seeking more information about ADHD from any source (Z=2.737; p=0.006), and having ever dealt with an ADHD patient (Z=3.023; p=0.002). On the other hand, a higher attitude score was more associated with the female gender (Z=2.574; p=0.010), having previously heard of ADHD (Z=5.813; p<0.001), familiarity with places for help-seeking ADHD (Z=2.887; p=0.004), and having ever dealt with an ADHD patient (Z=3.607; p<0.001). In contrast, unemployed participants were associated with lower attitude scores (H=13.110; p=0.001). No significant differences were observed between the knowledge and attitude scores in relation to age and education (p>0.05) (Table 5).

**Table 5:** Differences in the scores of knowledge and attitude in relation to the sociodemographic characteristics and practice toward ADHD (n=573) ^a^P-value has been calculated using the Mann-Whitney Z test. ^b^P-value has been calculated using Kruskal-Wallis H test. *Significant at p<0.05 level. ADHD, attention-deficit/hyperactivity disorder

Factor	Score (14)	Score (55)
	Mean ± SD	Mean ± SD
Age group^a^		
≤25 years	6.40 ± 1.37	39.9 ± 4.19
>25 years	6.61 ± 1.41	40.1 ± 4.19
Z-test; p-value	1.665; 0.096	0.025; 0.980
Gender^a^		
Male	6.49 ± 1.42	39.7 ± 4.18
Female	6.46 ± 1.32	40.5 ± 4.15
Z-test; p-value	0.250; 0.802	2.574; 0.010*
Occupation^b^		
Student	6.47 ± 1.46	40.3 ± 4.26
Employed	6.60 ± 1.36	40.4 ± 4.15
Unemployed	6.36 ± 1.31	39.1 ± 3.78
H-test; p-value	2.995; 0.224	13.110; 0.001*
Educational level^b^		
High school	6.47 ± 1.46	40.6 ± 4.06
College level	6.46 ± 1.35	39.8 ± 4.31
College degree or postgraduate	6.53 ± 1.41	39.9 ± 4.04
H-test; p-value	0.318; 0.853	3.510; 0.173
Previously heard ADHD^a^		
Yes	6.57 ± 1.37	40.6 ± 3.94
No	6.23 ± 1.39	38.2 ± 4.41
Z-test; p-value	2.261; 0.024*	5.813; <0.001*
Have you ever sought information about ADHD from any source?^a^		
Yes	6.62 ± 1.39	39.9 ± 4.45
No	6.32 ± 1.36	40.1 ± 3.84
Z-test; p-value	2.737; 0.006*	0.061; 0.951
Are you familiar with places that would help if you had a suspected ADHD child?^a^		
Yes	6.44 ± 1.36	40.5 ± 4.39
No	6.51 ± 1.40	39.7 ± 4.06
Z-test; p-value	0.506; 0.613	2.887; 0.004*
Have you ever dealt with an ADHD individual?^a^		
Yes	6.71 ± 1.39	40.5 ± 4.52
No	6.35 ± 1.33	39.6 ± 3.44
Z-test; p-value	3.023; 0.002*	3.607; <0.001*

## Discussion

This study assessed the KAP regarding ADHD among adults in Riyadh, Saudi Arabia. The results provide valuable insights into community-level understanding of ADHD and highlight areas where misconceptions persist.

Comparison with literature

Our findings showed that 75.2% of participants had heard of ADHD, a result consistent with the increasing global awareness reported by Polanczyk et al. [2]. However, relatively few participants recognized that ADHD could persist into adulthood, in contrast to the findings of Kessler et al., who reported that a large proportion of ADHD cases continue into adult life [5]. Regarding symptom recognition, 79.4% identified inattention and 77.1% identified hyperactivity as core symptoms, which aligns with Barkley and Barkley’s observations [8]. In contrast, only 42.4% recognized impulsivity, consistent with Asherson’s findings that impulsivity remains underrecognized despite being a diagnostic criterion [4]. Additionally, 18.2% of participants incorrectly identified aggression as a symptom, a misconception also documented among Saudi teachers by Munshi [6].

Etiological understanding was also mixed. 61.1% correctly identified genetic factors as a cause of ADHD, a finding consistent with Kessler et al. [5]. On the other hand, nearly 70% attributed ADHD to poor parenting or diet, misconceptions that have been similarly reported in previous Saudi studies [6,7]. With respect to treatment, 80.5% of participants acknowledged behavioral therapy, medication, and lifestyle modifications as effective management strategies, findings that parallel Alfageer et al., where the majority of teachers identified these approaches as beneficial [9]. Moreover, 85.2% of participants agreed that enhancing parenting skills could benefit children with ADHD, echoing Alanazi and Al Turki, who reported that 86.7% of teachers shared this view [7].

Attitudes toward ADHD were largely neutral to slightly positive, with a mean score of 39.9 (72.5%), comparable to results reported by Alfageer et al., who reported a mean score of 3.9 ± 0.40 [9]. These results suggest that while overtly negative attitudes are uncommon, a lack of strong positive attitudes reflects persistent uncertainty and stigma. In terms of practices, 55.7% of respondents sought ADHD-related information, with 78.7% relying on the internet, findings similar to those of Munshi, where 42.3% of teachers used the internet as their main source [6]. In our study, 17.6% reported using books as a source, compared with 32.8% in Alanazi and Al Turki [7]. Additionally, 43.6% of participants reported direct interaction with individuals diagnosed with ADHD, slightly lower than the 51.5% involvement reported by Alanazi and Al Turki among teachers [7].

Rationale and public health implications

The persistence of misconceptions in our sample may reflect limited national awareness campaigns and cultural stigma surrounding psychiatric conditions in Saudi Arabia. The positive correlation between higher knowledge and more favorable attitudes suggests that increasing public education could directly improve acceptance and reduce prejudice. The novelty of this study lies in its focus on the general adult population in Riyadh, whereas most previous Saudi studies have been limited to educators. Moreover, the use of a bilingual, online design enabled wide participation and inclusivity. These features provide new insights into the broader societal understanding of ADHD and emphasize the urgent need for public health interventions such as awareness campaigns, community-based training, and integration of ADHD education into healthcare and school systems.

Limitations

This study has several limitations. First, the cross-sectional design prevents causal inference, as associations between KAP cannot be interpreted as directional. Second, the online nature of data collection introduces sampling bias, favoring younger, educated, and technologically literate participants, while excluding individuals without internet access. This may have inflated the representation of more health-aware respondents, thereby underestimating the true knowledge gap in the general population. Third, reliance on self-reported questionnaires introduces the possibility of recall and social desirability bias, which may have led participants to overstate their awareness or attitudes. Fourth, cultural nuances in how ADHD is discussed or perceived may not have been fully captured in the survey design, potentially influencing responses. Finally, the study was conducted only in Riyadh, limiting generalizability to other regions of Saudi Arabia with differing sociocultural and educational contexts. These limitations highlight the need for future studies using mixed methods, larger nationally representative samples, and qualitative approaches to explore cultural influences on ADHD perceptions more deeply.

## Conclusions

This cross-sectional study among adults in Riyadh highlights that although general awareness of ADHD is relatively high, significant gaps persist regarding its causes, chronic course into adulthood, and recognition of less obvious symptoms such as impulsivity. Attitudes were largely neutral to moderately positive, with support for treatment and parenting interventions, yet persistent concerns regarding medication and school integration reflect ongoing stigma and misconceptions. Practical engagement with ADHD resources was limited, indicating insufficient translation of knowledge and attitudes into action. By addressing these gaps through culturally sensitive bilingual public education and targeted awareness initiatives, future efforts can enhance early recognition, reduce stigma, and strengthen community support for individuals living with ADHD.

## Appendices

Appendix A

1.       What is your gender?

·        A. Male

·        B. Female

2.      What is your occupation?

·        A. Student

·        B. Worker

·        C. Other

3.      What is your education level?

·        A. Primary school

·        B. Middle school

·        C. High school

·        D. College

·        E. Graduate

·        F. Postgraduate

4.      Have you ever heard of ADHD before?

·        A. Yes

·        B. No

5.      Do you believe children with ADHD can lead a normal life with proper treatment?

·        A. Strongly agree

·        B. Agree

·        C. Neutral

·        D. Disagree

·        E. Strongly disagree

6.      To what extent would you rely on an individual with ADHD to complete a critical task?

·        A. Completely

·        B. To some degree

·        C. Rarely

·        D. Not at all

7.      What are your emotional responses to your child consulting a psychiatrist for ADHD?

·        A. Worried

·        B. Neutral

·        C. Positive

8.      How would enhancement of parenting skills contribute to child well-being?

·        A. Not at all

·        B. Slightly

·        C. Moderately

·        D. Significantly

·        E. Very Significantly

9.      How beneficial is social skills training for children diagnosed with ADHD?

·        A. Not at all

·        B. Slightly

·        C. Moderately

·        D. Significantly

·        E. Very Significantly

10.    Should children with ADHD be treated differently at home?

·        A. Yes, different rules

·        B. No, same as others

·        C. More understanding and support

·        D. Depends on child

11.     To what extent do family issues contribute to ADHD?

·        A. Not at all

·        B. Slightly

·        C. Moderately

·        D. Significantly

·        E. Very Significantly

12.     Is ADHD due to lack of child effort to control behavior?

·        A. Strongly agree

·        B. Agree

·        C. Neutral

·        D. Disagree

·        E. Strongly disagree

13.    How might social media negatively impact a child's attention span?

·        A. Strongly agree

·        B. Agree

·        C. Neutral

·        D. Disagree

·        E. Strongly disagree

14.    Do ADHD medication side effects outweigh benefits?

·        A. Yes

·        B. No

·        C. Not sure

15.    Should children with ADHD attend regular or special schools?

·        A. Regular schools

·        B. Special schools

·        C. Not sure

16.    Would you feel uneasy if your child's classmate had ADHD?

·        A. Yes

·        B. No

·        C. Not sure

17.     Do you think medication is an effective treatment for ADHD?

·        A. Strongly agree

·        B. Agree

·        C. Neutral

·        D. Disagree

·        E. Strongly disagree

18.    Have you ever sought information about ADHD from any source?

·        A. Yes

·        B. No

19.    If yes, where? (Select all that apply)

·        A. Internet

·        B. Books

·        C. Healthcare professionals

·        D. Friends/Family

·        E. Media

·        F. Workshops/Seminars

·        G. I haven't sought information

20.    Are you familiar with places that help with ADHD diagnosis?

·        A. Yes

·        B. No

21.     If your child had ADHD, would you consider medication?

·        A. Yes

·        B. No

·        C. Not sure

22.    If yes, are you aware of medication side effects?

·        A. Yes

·        B. No

·        C. I didn't answer yes

23.    How often do you engage in ADHD learning activities?

·        A. Regularly

·        B. Occasionally

·        C. Rarely

·        D. Never

24.    Have you ever dealt with someone with ADHD?

·        A. Yes

·        B. No

·        C. Not sure

25.    If yes, who?

·        A. A relative

·        B. A friend

·        C. At work

·        D. I didn't answer yes

26.    Do you know how to advocate for a child with ADHD at school?

·        A. Yes

·        B. No

27. Are you from Riyadh?

·        A. Yes

·        B. No
